# Identification of PSMD14 as a potential novel prognosis biomarker and therapeutic target for osteosarcoma

**DOI:** 10.1002/cnr2.1522

**Published:** 2021-08-12

**Authors:** Yubao Gong, Zheng‐Ren Wei

**Affiliations:** ^1^ Department of Orthopedics Jilin University First Hospital Jilin China; ^2^ Department of Pharmocology Jilin University Bethune College of Medicine Jilin China

**Keywords:** biomarker, differential expressed genes, gene set enrichment analysis, metastasis, osteosarcoma, prognosis, PSMD14

## Abstract

**Background:**

Osteosarcoma is the most common primary bone tumor. The survival rate of osteosarcoma patients has not significantly increased in the past decades. Uncovering the mechanisms of malignancy, progression, and metastasis will shed light on the development of new therapeutic targets and treatment for osteosarcoma.

**Aim:**

The aim of this study is to identify potential osteosarcoma biomarker and/or therapeutic targets by using integrated bioinformatics analysis.

**Methods and results:**

We utilized existing gene expression datasets to identify differential expressed genes (DEGs) that could serve as osteosarcoma biomarkers or even as therapeutic targets. We found 48 DEGs were overlapped in three datasets. Among these 48 DEGs, PSMD14 was on the top of the up‐regulated gene list. We further found that higher PSMD14 expression was correlated with higher risk group (younger age group, ≤20.83 years of age), metastasis within 5 years and higher grade of tumor. Higher PSMD14 expression in osteosarcoma had positive correlation with higher infiltration of CD8+ T cells, neutrophils and myeloid dendritic cells. Kaplan‐Myer survival data further revealed that higher expression of PSMD14 predicted significantly worse prognosis (*p* = .013). Gene set enrichment analysis was further performed for the DEGs related to PSMD14 in osteosarcoma. We found that lower PSMD14 expression group had more immune responses such as interferon γ, α responses, inflammation response etc. However, the higher PSMD14 expression group had more cell proliferation‐related biological processes, such as G2M checkpoints and Myc targets. Through establishing protein–protein interaction networks using PSMD14 related DEGs, we identified 10 hub genes that were all ribosomal proteins. These hub genes may play roles in osteosarcoma tumorigenesis, progression and/or metastasis.

**Conclusion:**

We identified PSMD14 gene as a possible osteosarcoma biomarker, and/or a possible therapeutic target.

## 1 BACKGROUND

Osteosarcoma is the most common primary bone tumor.^1^ It mostly occurs in children and young adults. Although osteosarcoma can develop at any age, teenagers are the most commonly affected age group.^1^ It occurs to more than 3.4 million people worldwide every year.^2^ Great advances have been made in the treatment and management of osteosarcoma in the recent years, but a significant percentage of patients do not survive because of numerous reasons, such as metastasis.^3^ The 10‐year survival rate for patients with metastatic osteosarcoma is only 24.0% compared to 65.8% for the patients with local/regional disease.^3^

Although by developing target therapies has significantly increased the survival rate of other cancers, the survival rate of osteosarcoma patients has remained the same in the recent a few decades.^4^ Understanding the mechanisms of the malignancy, finding the prognosis markers and potential therapeutic target(s) are the keys to significantly increasing the survival rate after treatment. Although the mechanisms of osteosarcoma malignancy is far from fully understood, alteration of gene expression has been known to play significant roles in the tumorigenesis of osteosarcoma.^4^ Several gene alterations have been identified to be associated with osteosarcoma, such as TP53,^5^, ^6^, ^7^ Rb,^8^ Myc.^9^ Recently gene expression profiling was used to uncover the pathophysiology. These gene expression datasets provided insight to the gene expression alteration in osteosarcoma. Comparing multiple gene expression datasets of osteosarcoma will provide further information for potential prognosis markers or therapeutic targets. In this study, we utilized multiple datasets to identify potential osteosarcoma prognosis markers and/or therapeutic targets.

As a component of 26S proteasome, PSMD14 forms multiprotein complex with several other components in the proteasome. Together, they are involved in the ATP‐dependent degradation of ubiquitinated proteins.^10^, ^11^, ^12^ This is the most important machinery to maintenance of protein homeostasis by removing misfolded or damaged proteins. Besides involved in the proteasome function, PSMD14 reportedly plays critical roles in regulating many signaling pathways, such as double‐strand DNA break repairs,^10^ embryonic stem cell differentiation,^13^ cellular proliferation,^14^, ^15^ maintenance of protein stability,^16^, ^17^ Golgi‐to‐ER retrograde transport,^18^ and inflammasome activation.^19^ However, whether PSMD14 plays any roles in osteosarcoma is not elucidated. In this study, we utilized existing osteosarcoma gene expression profile datasets from Gene Expression Ominbus (GEO) and identified PSMD14 as a top differential expressed gene (DEG). Based on the novelty and our preliminary screens results, we did further analysis on the correlation of PSMD14 expression and osteosarcoma clinical features. The expression level of PSMD14 was found to be highly correlated to osteosarcoma prognosis. This finding hinted that PSMD14 could potentially be used as an osteosarcoma prognosis marker, could also potentially be used as a therapeutic target.

## 2 MATERIALS AND METHODS

### 2.1 Gene expression profile datasets

Five gene expression profile datasets were downloaded (https://www.ncbi.nlm.nih.gov/geo) and analyzed in this study, including GSE14359, GSE42352, GSE39262, TCGA‐SARC and TARGET‐OS. GSE14359 gene expression dataset was using osteosarcoma tissues (GSM359139, GSM359140, GSM359143, GSM359144, GSM359147, GSM359148, GSM359149, GSM359150, GSM359155 and GSM359156) and two non‐neoplastic primary osteoblast cell lines as controls (GSM359137 and GSM359138).^20^ Microarray was performed on GPL96 chip. GSE42352 dataset used 84 pre‐treatment osteosarcoma biopsies (GSM825626–GSM825709), and 12 mesenchymal stem cell lines or three osteoblast cell lines as normal cell controls (GSM717846–GSM717857).^21^, ^22^, ^23^ GSE39262 dataset was comparing two primary untransformed cell lines (GSM959039 and GSM959040) with 10 osteosarcoma cell lines (GSM958993–GSM959002). TCGA‐SARC dataset included 261 osteosarcoma patients with 55.6% of them were older than 60 years and 54.4% of them were females. TATGET‐OS dataset included 88 osteosarcoma patients.

### 2.2 Identification of differential expressed genes

DEGs between the osteosarcoma samples and control samples in all datasets were screened by using the Limma package.^24^ Criteria used for DEGs are *p* < .05 and “fold change” >1.5. The overlapping of the DEGs between the datasets identified from Limma analysis of each dataset were analyzed with VENN package and plotted with VennDiagram.^25^ For identification of DEGs associated with PSMD14 expression, osteosarcoma patients were divided into high PSMD14 expression and low PSMD14 expression groups by using median expression of PSMD14 as cut‐off.

### 2.3 Gene ontology enrichment assay

Gene ontology enrichment of the DEGs were analyzed by using Metascape (https://metascape.org) online software,^26^ with the parameters setting as follows: min overlap = 3, min enrichment = 1.5 and *p* value cut‐off = .01. Biological Process, Molecular Function, Cellular Component categories were focused and presented.

### 2.4 Protein–protein interaction network analysis

In order to understand the interactions among the DEGs in osteosarcoma, we used STRING (http://www.string-db.org)^27^ to predict the potential interactions (score > 0.4). The interaction networks were visualized by using Cytoscape software.^28^ PPI network within the proteins that potentially interact with DEG proteins were analyzed and established by using NetworkAnalyst (https://www.networkanalyst.ca) online analysis.^29^ PPI networks within the DEGS associated to PMSD14 expression were analyzed by using MCODE, and top 10 hub genes were identified by using CytoHubba.^30^

### 2.5 Immune cells infiltration analysis

Immune cells infiltration in osteosarcoma tissues analysis was performed by analyzing the TCGA gene expression profile dataset and TARGET dataset on TIMER2.0 web server.^31^ The correlation between expression of PSMD14 gene and the immune cells infiltration was analyzed.

### 2.6 Survival analysis

The overall effect of the expression level of PSMD14 on patient survival was analyzed by using Kaplan–Meier curve. Patients in the dataset obtained from TARGET were divided into PSMD14 high expression group and PSMD14 low expression group by using median expression of PSMD14 as cut‐off. The difference between the low and high PSMD14 expression groups was analyzed by log‐rank test. A P value less than 0.05 was defined as significant different.

### 2.7 Gene set enrichment analysis

In order to analyze the gene ontology of the genes significantly correlated to PSMD14 expression in osteosarcoma, we utilized gene set enrichment analysis (GSEA) computational method.^32^ Patients were divided into PSMD14 high‐expression and low‐expression groups by using median expression of PSMD14 as cutoff. The gene sets (h.all.v7.1.symbols.gmt) used in GSEA analysis were obtained from the MSigDB database (Molecular Signatures Databases, http://software.broadinstitute.org/gsea/downloads.jsp). Gene set permutations were performed 1000 times for each analysis and the permutation type was set to “Gene_set.” The statistically significant pathways enrichment was defined as FDR <0.05. The Normalized Enrichment Score (NES) was used to measure the gene‐sets enrichment.

### 2.8 Statistical analysis

R programme (V3.4.3) was used for all statistical analysis. Logistic regression model was use to analyze the correlation between the expression of PSMD14 and clinical characterizations. Two‐tailed Student's *T*‐test was also used for comparison of two groups. *p* Value less than .05 was considered as statistically significant.

## 3 RESULTS

Identification of DEGs in osteosarcoma and gene ontology analysis.

**FIGURE 1:**
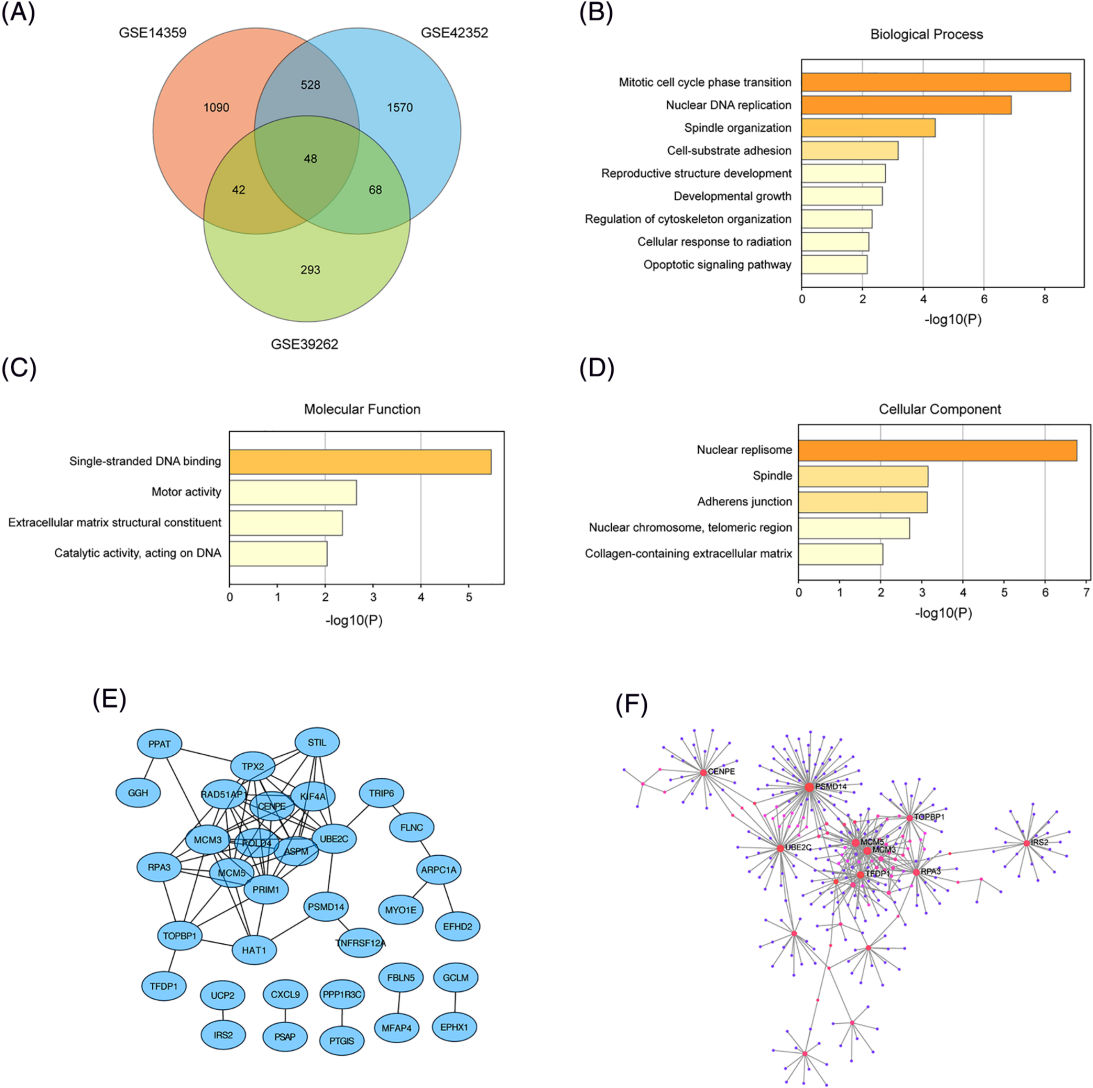
Identification of differential expressed genes (DEGs) in osteosarcoma and analysis of their function. (A) Vann diagram showed numbers of significant DEGs derived from GSE14359, GSE42352, and GSE39262 3 datasets. (B) Gene ontology analysis of DEGs showed over represented biological process categories. (C) Gene ontology analysis of DEGs showed over represented molecular function categories. (D) Gene ontology analysis of DEGs showed over represented cellular component categories. (E) Protein–protein‐interaction network of DEGs. (F) PPI network constructed from proteins coded by DEGs and the proteins they interact with

In order to identify the DEGs in osteosarcoma, we analyzed three genome‐wide gene expression profile datasets GSE14359,^20^ GSE42352,^21^, ^22^ and GSE39262. From these three datasets, we identified 1708, 2214 and 451 DEGs, respectively, in osteosarcoma to compared normal (or non‐neoplastic) cells (Figure 1A). There are 48 DEGs were overlapping within the DEGs from the three datasets (Figure 1A). To understand the overall function of the overlapped DEGs, we further performed gene ontology enrichment analysis for these 48 genes, focusing on three categories, biological process, molecular function and cellular component. We found that these genes were enriched in these biological process categories: mitotic cell cycle phase transition, nuclear DNA replication, spindle organization (Figure 1B). In molecular function category, these genes were mainly enriched in single‐stranded DNA binding, motor activity (Figure 1C). In cellular component category, we found nuclear replisome was the most enriched category (Figure 1D). To understand the relationship between the proteins encoded by these DEGs, we performed protein–protein interaction (PPI) analysis, we found that 25 proteins among the proteins encoded by these DEGs interact with each other (Figure 1E). Furthermore, numerous proteins are predicted to interact with these proteins and form a PPI network (Figure 1F). The results indicated that these DEGs may play important roles in DNA replication and cell proliferation.

### 3.1 Up‐regulation of PSMD14 expression was correlated to osteosarcoma

**FIGURE 2:**
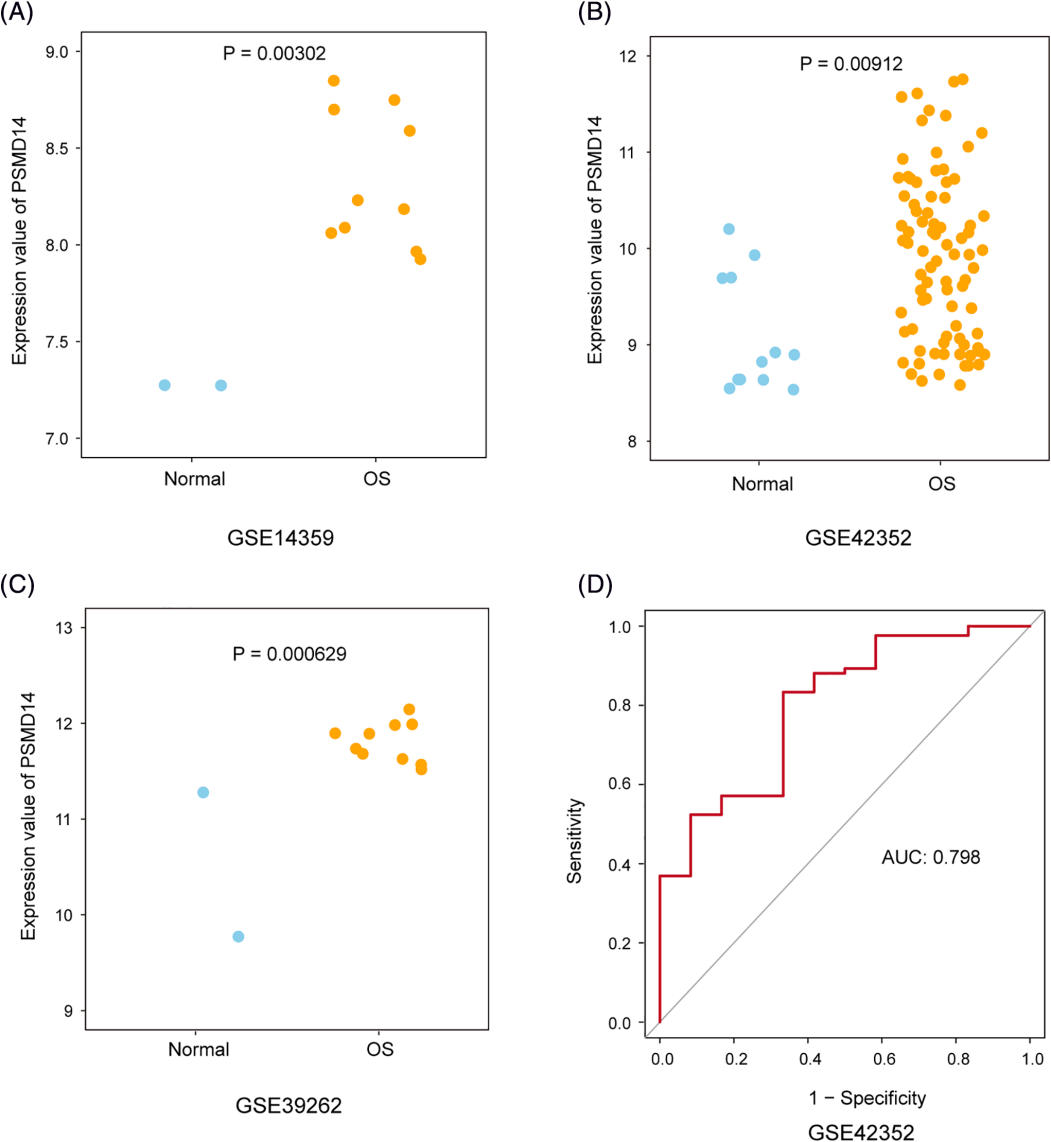
PSMD14 expression was up regulated in osteosarcoma compare with normal tissue. (A) PSMD14 expression was up regulated in osteosarcoma compare with normal tissue in GSE14359 dataset. (B) PSMD14 expression was up regulated in osteosarcoma compare with normal tissue in GSE42352 dataset. (C) PSMD14 expression was up regulated in osteosarcoma compare with normal tissue in GSE39262 dataset. (D) A receiver operating characteristic (ROC) curve plotted with data from GSE42352 dataset showed that PSMD14 expression could positively predict whether the tissue was osteosarcoma

Among the 48 DEGs, Table 1 listed top 10 up‐regulated and Table 2 listed top 10 down‐regulated genes. Among the top 10 up‐regulated genes, we found that PSMD14 was the most significantly up‐regulated gene. PSMD14 has been recently reported to play important roles in other cancers, such as lung adenocarcinoma,^14^, ^33^ hepatocellular carcinoma,^34^, ^35^ glioma,^17^ and esophageal squamous cell carcinoma.^36^ But whether PSMD14 plays any roles in osteosarcoma is not studied. Based on the preliminary screening analysis and the novelty, we performed further analysis focus on PSMD14 gene. In all three datasets, PSMD14 gene was significantly up‐regulated (Figure 2A–C). By using relative larger sample sized GSE42352 dataset, the receiver operating characteristic (ROC) showed that PSMD14 expression could positively predict whether the tissue was osteosarcoma (Figure 2D). This finding hinted a possibility of using PSMD14 as a diagnosis marker for osteosarcoma.

**TABLE 1:** Top 10 up‐regulated genes within 48 overlapped differential expressed genes

Gene_symbol	LogFC	AveExpr	*t*	*p* Value	Adj. *p* Val	*B*
PSMD14	1.427325868	9.913164214	14.17274684	7.23E‐24	1.81E‐19	43.67537197
LOC642989	2.980752783	9.915280739	11.99730004	8.21E‐20	1.03E‐15	34.58877066
CIR	0.931698515	8.365467052	11.71229554	2.90E‐19	2.42E‐15	33.35670089
LOC647037	1.116746869	8.60000568	11.12889604	3.94E‐18	2.46E‐14	30.808767
LOC648638	1.195739225	8.769178095	11.05467066	5.51E‐18	2.75E‐14	30.48227439
C14orf166	1.27363133	10.46655663	10.88810191	1.17E‐17	4.86E‐14	29.74782785
LOC401206	2.689100901	12.0816709	10.83329429	1.50E‐17	5.33E‐14	29.50564865
LOC649682	2.779189943	10.75498554	10.80398935	1.71E‐17	5.33E‐14	29.37605638
FTHL2	2.418951668	10.31390646	10.75856883	2.10E‐17	5.82E‐14	29.17505926

**TABLE 2:** Top 10 down‐regulated genes within 48 overlapped differential expressed genes

Gene_symbol	LogFC	AveExpr	*t*	*p* Value	Adj. *p* Val	*B*
ANXA5	−0.88915948	13.22046799	−8.03301762	5.62E‐12	3.80E‐10	16.94555225
TOR3A	−0.6897186	9.610793107	−7.59733629	4.12E‐11	2.02E‐09	14.99893603
ZNF364	−0.73443752	10.39599484	−7.34070142	1.32E‐10	5.70E‐09	13.86039414
EIF2B1	−0.59002216	9.373840845	−7.21860446	2.29E‐10	9.12E‐09	13.32138442
TOB1	−0.91197591	9.003275488	−7.00111431	6.10E‐10	2.16E‐08	12.36620444
FLJ20487	−0.67582273	10.35189531	−6.93159119	8.33E‐10	2.83E‐08	12.06234363
LOC651348	−0.80449704	8.395034784	−6.58526024	3.88E‐09	1.09E‐07	10.56086426
C5orf15	−0.70006481	11.66152439	−6.56416491	4.26E‐09	1.18E‐07	10.4701259
ADCK2	−0.60479266	9.698811124	−6.31788359	1.26E‐08	3.10E‐07	9.417640489
HLA‐DMA	−1.37998955	11.63848864	−6.16888881	2.40E‐08	5.45E‐07	8.787521116

### 3.2 High expression of PSMD14 gene was correlated to osteosarcoma clinical features

**FIGURE 3:**
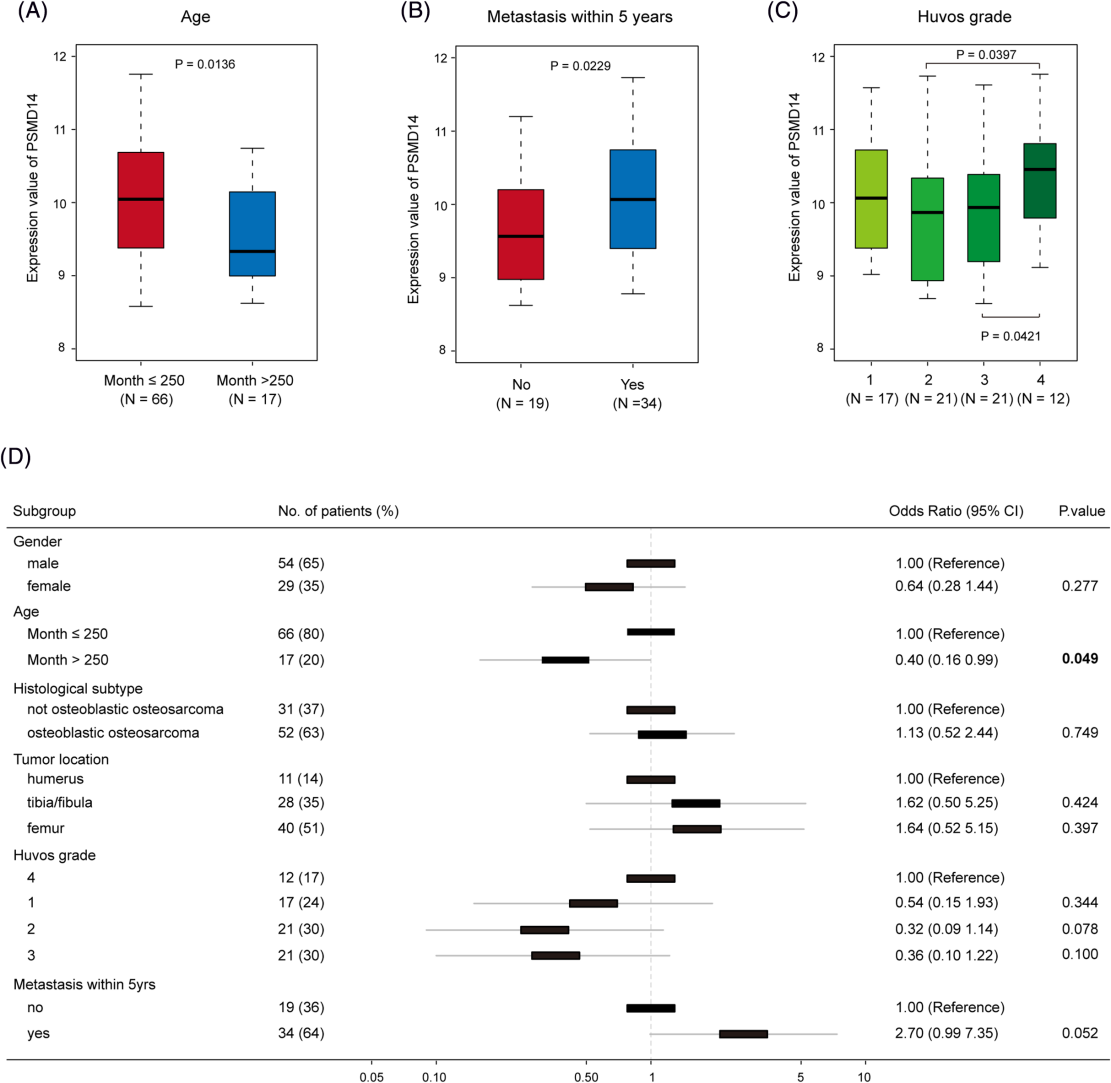
PSMD14 expression was correlated to osteosarcoma clinical features. (A) Higher PSMD14 expression was positively correlated to higher risk age group (≤20.83 years old), *p* = .0136. (B) PSMD14 expression was higher in patients had metastatic osteosarcoma in 5 years compare with those who did not have metastasis. (C) PSMD14 expression level comparison between the different osteosarcoma Huvos grades. Grade 4 osteosarcoma exhibited higher PSMD14 expression compared with grade 2 (*p* = .0397) and grade 3 osteosarcoma (*p* = .0421). (D) Correlation of PSMD14 expression to the clinical characters of osteosarcoma patients

In order to further understand whether high expression of PSMD14 in osteosarcoma is correlated to clinical features, we analyzed the correlation of PSMD14 expression level and osteosarcoma patients' clinical data from dataset GSE42352. The results indicated that younger patients (≤20.83 years old) expressed higher level of PSMD14 compare to older patients (>20.83 years old; Figure 3A, *p* = .0136). Patients who progressed to metastasis within 5 years expressed higher PSMD14 compare to those without metastasis (Figure 3B, *p* = .0229). This result indicate that PSMD14 expression could be a potential prognosis marker. When we compared the PSMD14 gene expression level between the patients with different Huvos Grade of osteosarcoma, we found that higher grade, that is, grade 4 had higher level of PSMD14 expression compare to grade 2 and grade 3 (Figure 3C). Logic regression model analysis further confirmed that age of the patients was significantly correlated to PSMD14 gene expression level (Figure 3D).

### 3.3 High expression of PMSD14 gene was correlated to osteosarcoma immune cell infiltration and poor prognosis

**FIGURE 4:**
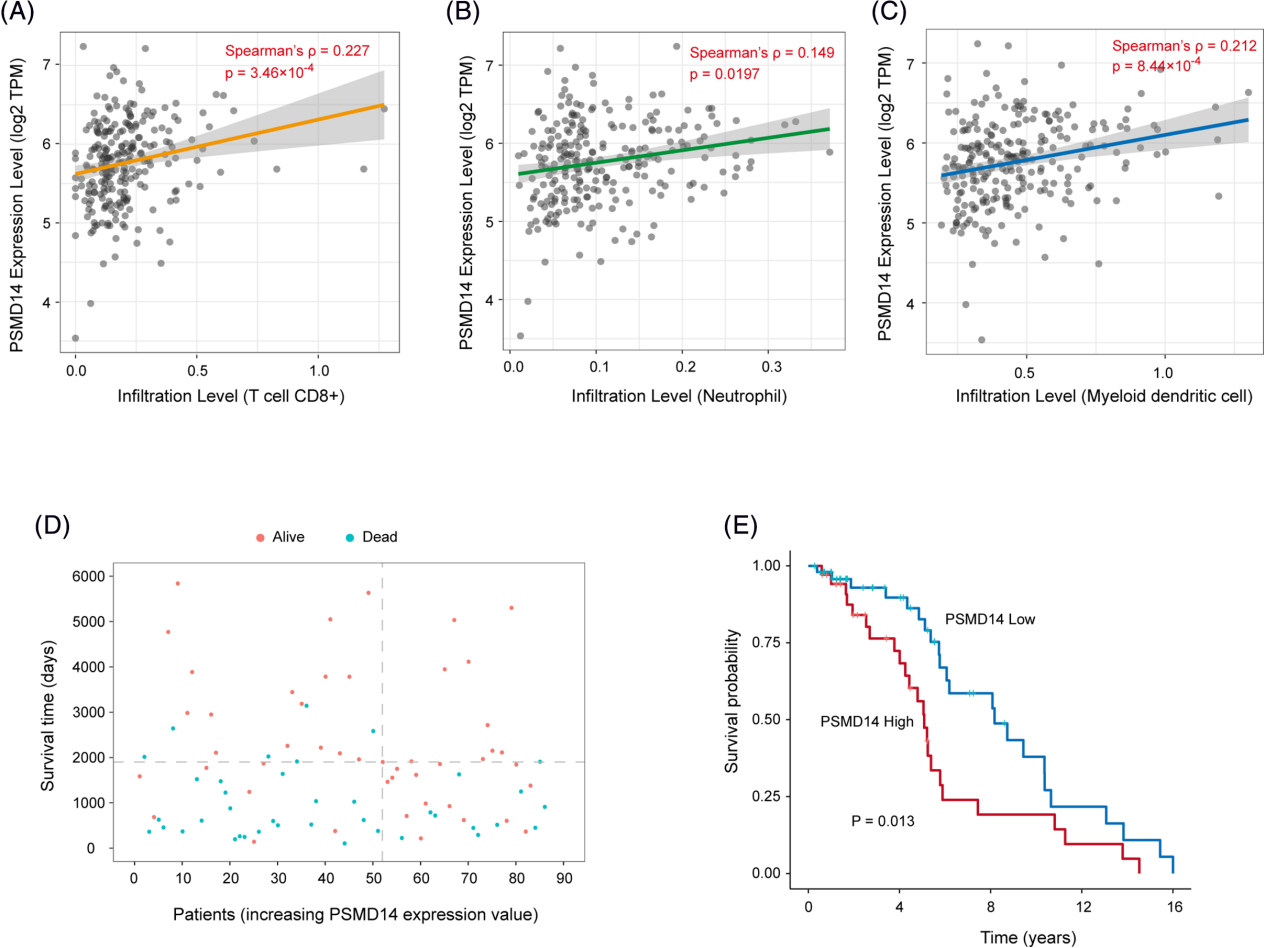
Higher PSMD14 expression was correlated to immune cells infiltration and worse prognosis. (A) Positive correlation of PSMD14 expression and CD8+ T cells infiltration in osteosarcoma. (B) Positive correlation of PSMD14 expression and neutrophil infiltration in osteosarcoma. (C) Positive correlation of PSMD14 expression and myeloid dendritic cells in osteosarcoma. (D) Correlation of PSMD14 expression and patient survival. Data analyzed from osteosarcoma patients in TARGET dataset. (E) Kaplan–Meier curve of PSMD14 high and low expression groups (*p* = .013)

By analyzing the SARC patients gene profile dataset from the TCGA, we found that expression level of PSMD14 was positively correlated to infiltration of CD8+ T lymphocytes (Figure 4A), neutrophils (Figure 4B), as well as myeloid dendritic cells (Figure 4C). We further analyzed osteosarcoma patient dataset from TARGET database by comparing PSMD14 high expression patient group to PSMD14 low expression patient group. The result indicated that higher PSMD14 expression was correlated to shorter survival time (Figure 4D). The Kaplan–Meier curve further showed that high expression of PSMD14 was significantly correlated to poor prognosis (Figure 4E).

### 3.4 Gene ontology analysis of DEGs associated with expression level of PSMD14 gene

**FIGURE 5:**
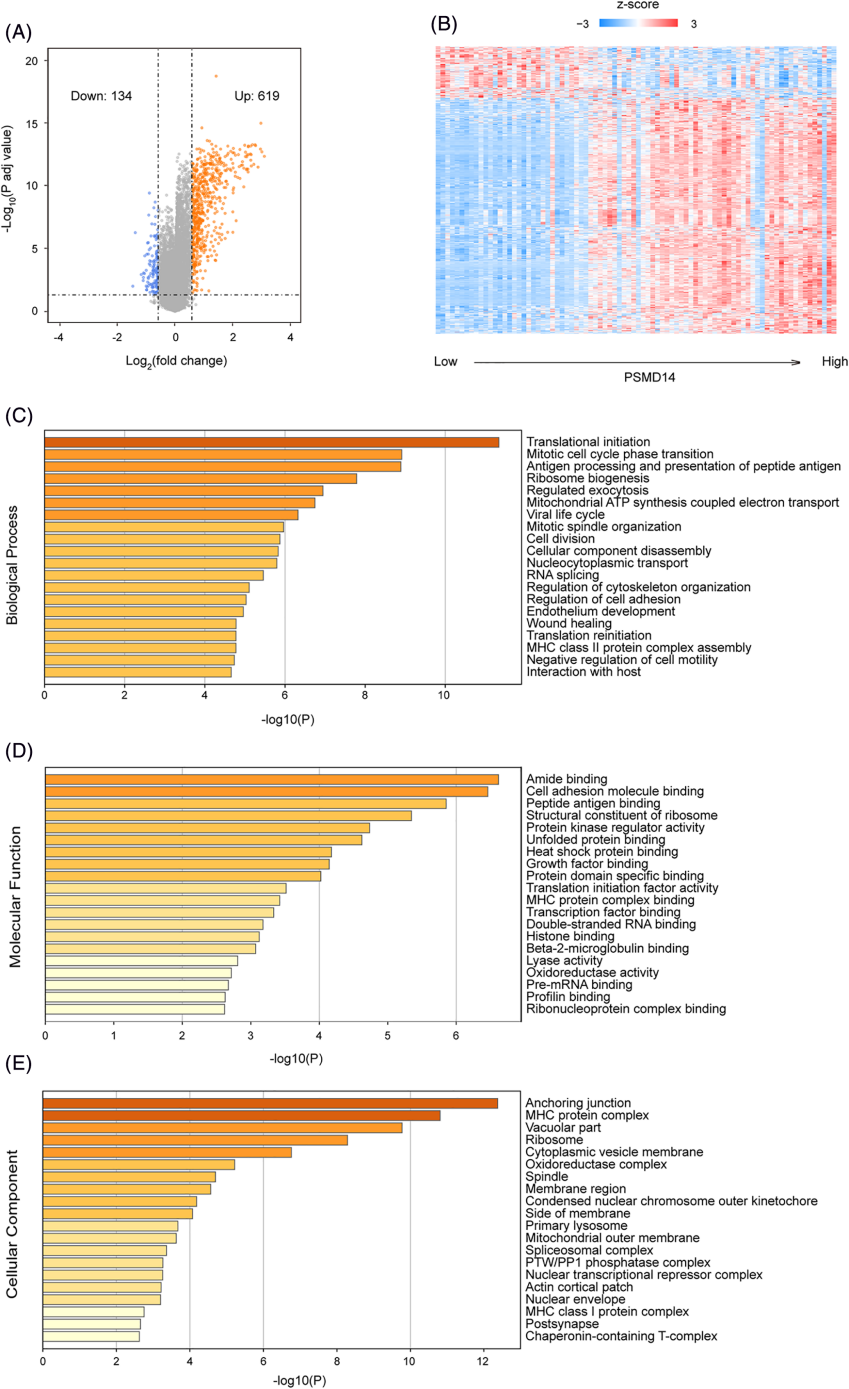
Identification of significant differential expressed genes (DEGs) in osteosarcoma between PSMD14 high and low expression groups and analysis of their function. (A) Volcano plot of significant DEGs between PSMD14 high and low expression groups to identify up and down regulated genes. In PSMD14 high expression group, 619 genes were found up‐regulated and 134 genes were found down‐regulated. (B) Heat map showed the relative expression of DEGs in each patients. (C) DEGs Gene Ontology analysis showed categories enriched in Biological Processes. (D) DEGs Gene Ontology analysis showed categories enriched in Molecular Function. (E) DEGs Gene Ontology analysis showed categories enriched in Cellular Component

We utilized GSE42352 dataset to identify genes that were differentially expressed between the high and low PSMD14 expression groups in osteosarcoma, by dividing the GSE42352 cohort into PSMD14 high expression and PSMD14 low expression groups based on the median expression level of PSMD14. The characteristics of the patients are listed in Table 3. We identified 753 DEGs, including 619 genes that were significantly up‐regulated in PSMD14 high group, and 134 genes that were significantly down regulated in the PSMD14 high group (Figure 5A,B). Gene ontology enrichment analysis (Tables 4, 5, 6) further revealed that these DEGs were overrepresented in translational initiation, mitotic cell cycle phase transition, antigen processing and presentation of peptide antigen, and ribosome biogenesis categories (Figure 5C, Table 4). For molecular function, these DEGs were overrepresented in amide binding, cell adhesion molecule binding, peptide antigen binding, structural constituent of ribosome, and protein kinase regulator activity categories (Figure 5D, Table 5). As for cellular component terms, genes were primarily enriched in anchoring junction, MHC protein complex, vacuolar part, ribosome, and cytoplasmic vesicle membrane (Figure 5E, Table 6).

**TABLE 3:** Characteristics of patients

Characteristics	Osteosarcoma (*n* = 83)		
	Total	Low PMSD14 expression	High PMSD14 expression
Age (month)	192 (37–948)	200 (37–696)	181 (81–948)
Gender			
Male	54 (65.1)	23 (59.0)	31 (70.5)
Female	29 (34.9)	16 (41.0)	13 (29.5)
Huvos grade			
1	17 (20.5)	6 (15.4)	11 (25.0)
2	21 (25.3)	11 (28.2)	10 (22.7)
3	21 (25.3)	10 (25.6)	11 (25.0)
4	12 (14.5)	3 (7.7)	9 (20.5)
Unknown	12 (14.5)	9 (23.1)	3 (6.8)
Metastasis within 5 years			
Yes	34 (41.0)	14 (35.9)	20 (45.5)
No	19 (22.9)	12 (30.8)	7 (15.9)
Unknown	30 (36.1)	13 (33.3)	17 (38.6)

**TABLE 4:** List of enriched Gene Ontology categories in Biological Processes

GO	Description	Count	%	Log10 (*p*)	Log10 (*q*)
GO:0006413	Translational initiation	26	4.2	−11.34	−7.14
GO:0044772	Mitotic cell cycle phase transition	43	6.95	−8.91	−5.47
GO:0048002	Antigen processing and presentation of peptide antigen	23	3.72	−8.89	−5.47
GO:0042254	Ribosome biogenesis	27	4.36	−7.79	−4.74
GO:0045055	Regulated exocytosis	47	7.59	−6.95	−4.08
GO:0042775	Mitochondrial ATP synthesis coupled electron transport	14	2.26	−6.75	−3.93
GO:0019058	Viral life cycle	26	4.2	−6.33	−3.63
GO:0007052	Mitotic spindle organization	14	2.26	−5.96	−3.36
GO:0051301	Cell division	37	5.98	−5.87	−3.32
GO:0022411	Cellular component disassembly	35	5.65	−5.83	−3.3
GO:0006913	Nucleocytoplasmic transport	26	4.2	−5.8	−3.28
GO:0008380	RNA splicing	29	4.68	−5.46	−3.04
GO:0051493	Regulation of cytoskeleton organization	33	5.33	−5.11	−2.74
GO:0030155	Regulation of cell adhesion	39	6.3	−5.03	−2.7
GO:0003158	Endothelium development	14	2.26	−4.96	−2.66
GO:0042060	Wound healing	33	5.33	−4.78	−2.53
GO:0002399	MHC class II protein complex assembly	3	0.48	−4.78	−2.53
GO:0002188	Translation reinitiation	3	0.48	−4.78	−2.53
GO:2000146	Negative regulation of cell motility	24	3.88	−4.73	−2.5
GO:0051701	Interaction with host	17	2.75	−4.66	−2.45

**TABLE 5:** List of enriched Gene Ontology categories in Molecular Function

GO	Description	Count	%	Log10 (*p*)	Log10 (*q*)
GO:0033218	Amide binding	29	4.68	−6.62	−3.27
GO:0050839	Cell adhesion molecule binding	34	5.49	−6.46	−3.27
GO:0042605	Peptide antigen binding	8	1.29	−5.85	−2.79
GO:0003735	Structural constituent of ribosome	16	2.58	−5.35	−2.38
GO:0019887	Protein kinase regulator activity	16	2.58	−4.73	−1.93
GO:0051082	Unfolded protein binding	13	2.1	−4.62	−1.91
GO:0031072	Heat shock protein binding	12	1.94	−4.18	−1.56
GO:0019838	Growth factor binding	13	2.1	−4.15	−1.56
GO:0019904	Protein domain specific binding	36	5.82	−4.02	−1.53
GO:0003743	Translation initiation factor activity	7	1.13	−3.52	−1.13
GO:0023023	MHC protein complex binding	5	0.81	−3.42	−1.08
GO:0008134	Transcription factor binding	32	5.17	−3.33	−1.01
GO:0003725	Double‐stranded RNA binding	8	1.29	−3.18	−0.89
GO:0042393	Histone binding	14	2.26	−3.12	−0.86
GO:0030881	Beta‐2‐microglobulin binding	3	0.48	−3.07	−0.82
GO:0016829	Lyase activity	13	2.1	−2.81	−0.59
GO:0016491	Oxidoreductase activity	33	5.33	−2.71	−0.51
GO:0036002	Pre‐mRNA binding	5	0.81	−2.67	−0.49
GO:0005522	Profilin binding	3	0.48	−2.63	−0.46
GO:0043021	Ribonucleoprotein complex binding	10	1.62	−2.62	−0.46

**TABLE 6:** List of enriched Gene Ontology categories in Cellular Components

GO	Description	Count	%	Log10 (*p*)	Log10 (*q*)
GO:0070161	Anchoring junction	48	7.75	−12.37	−9.37
GO:0042611	MHC protein complex	11	1.78	−10.81	−8.3
GO:0044437	Vacuolar part	44	7.11	−9.77	−7.33
GO:0005840	Ribosome	24	3.88	−8.29	−6.18
GO:0030659	Cytoplasmic vesicle membrane	46	7.43	−6.76	−4.87
GO:1990204	Oxidoreductase complex	13	2.1	−5.22	−3.47
GO:0005819	Spindle	24	3.88	−4.69	−3
GO:0098589	Membrane region	23	3.72	−4.57	−2.91
GO:0000942	Condensed nuclear chromosome outer kinetochore	3	0.48	−4.18	−2.61
GO:0098552	Side of membrane	32	5.17	−4.07	−2.52
GO:0005766	Primary lysosome	13	2.1	−3.67	−2.18
GO:0005741	Mitochondrial outer membrane	14	2.26	−3.63	−2.15
GO:0005681	Spliceosomal complex	14	2.26	−3.36	−1.93
GO:0072357	PTW/PP1 phosphatase complex	3	0.48	−3.27	−1.85
GO:0090568	Nuclear transcriptional repressor complex	5	0.81	−3.26	−1.85
GO:0030479	Actin cortical patch	4	0.65	−3.22	−1.83
GO:0005635	Nuclear envelope	25	4.04	−3.2	−1.82
GO:0042612	MHC class I protein complex	3	0.48	−2.76	−1.42
GO:0098794	Postsynapse	29	4.68	−2.66	−1.33
GO:0005832	Chaperonin‐containing T‐complex	3	0.48	−2.63	−1.31

### 3.5 Gene set enrichment analysis

**FIGURE 6:**
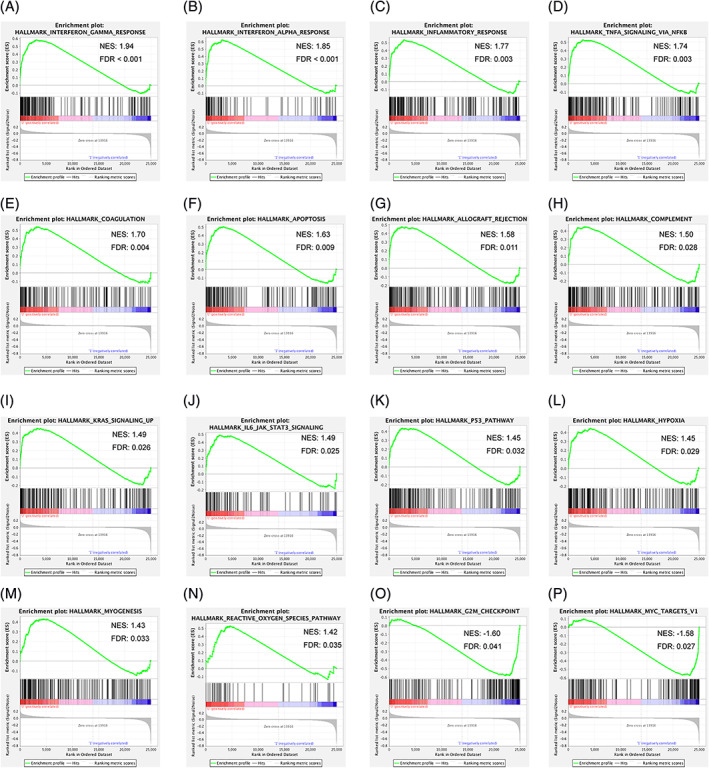
Gene Set Enrichment Analysis (GSEA) of significant differential expressed genes. (A–N) Enriched biological states or processes identified by GSEA in PSMD14 low expression group. (O and P) Enriched biological states or processes identified by GSEA in PSMD14 high expression group

GSEA verified that enriched biological processes were different, when comparing PSMD14 low expression group with PSMD14 high expression group (Table 7). Significantly enriched biological process in the PSMD14 low expression group included: interferon γ response (Figure 6A), interferon α response (Figure 6B), inflammatory response (Figure 6C), TNFa signaling via NF‐κB (Figure 6D), coagulation (Figure 6E), Apoptosis (Figure 6F), allograft rejection (Figure 6G), complement (Figure 6H), Kras signaling (Figure 6I), IL‐6/JAK/STAT3 signaling (Figure 6J), P53 pathway (Figure 6K), hypoxia (Figure 6L), myogenesis (Figure 6M), and reactive oxygen species pathway (Figure 6N). However, in the PSMD14 high expression group, enriched categories were more related to cell proliferation, such as G2M checkpoint (Figure 6O) and Myc target (Figure 6P). This data indicated that low PSMD14 expression osteosarcoma patients may have higher immune response.

**TABLE 7:** Gene set enrichment analysis of DEGs between PSMD14 high and low expression groups

HALLMARK_ID	Size	ES	NES	NOM *p*‐val	FDR *q*‐val	FWER *p*‐val	Rank	Leading edge
HALLMARK_INTERFERON_GAMMA_RESPONSE	182	0.58	1.94	<0.001	<0.001	<0.001	3078	Tags = 48%, List = 12%, Signal = 55%
HALLMARK_INTERFERON_ALPHA_RESPONSE	83	0.62	1.85	<0.001	<0.001	<0.001	3117	Tags = 57%, List = 12%, Signal = 64%
HALLMARK_INFLAMMATORY_RESPONSE	187	0.54	1.77	<0.001	0.003	0.004	2899	Tags = 39%, List = 12%, Signal = 43%
HALLMARK_TNFA_SIGNALING_VIA_NFKB	182	0.53	1.74	<0.001	0.003	0.005	2732	Tags = 37%, List = 11%, Signal = 41%
HALLMARK_COAGULATION	134	0.54	1.7	<0.001	0.004	0.009	3150	Tags = 34%, List = 13%, Signal = 38%
HALLMARK_APOPTOSIS	153	0.5	1.63	<0.001	0.009	0.021	3405	Tags = 42%, List = 14%, Signal = 48%
HALLMARK_ALLOGRAFT_REJECTION	183	0.48	1.58	<0.001	0.011	0.03	2432	Tags = 30%, List = 10%, Signal = 32%
HALLMARK_COMPLEMENT	188	0.45	1.5	<0.001	0.028	0.09	3106	Tags = 36%, List = 12%, Signal = 40%
HALLMARK_KRAS_SIGNALING_UP	182	0.45	1.49	<0.001	0.026	0.093	3326	Tags = 35%, List = 13%, Signal = 40%
HALLMARK_IL6_JAK_STAT3_SIGNALING	85	0.49	1.49	0.007	0.025	0.098	2674	Tags = 38%, List = 11%, Signal = 42%
HALLMARK_P53_PATHWAY	173	0.44	1.45	<0.001	0.032	0.139	2553	Tags = 32%, List = 10%, Signal = 35%
HALLMARK_HYPOXIA	180	0.45	1.45	<0.001	0.029	0.139	4232	Tags = 41%, List = 17%, Signal = 48%
HALLMARK_MYOGENESIS	187	0.43	1.43	<0.001	0.033	0.166	4493	Tags = 35%, List = 18%, Signal = 42%
HALLMARK_REACTIVE_OXYGEN_SPECIES_PATHWAY	43	0.53	1.42	0.056	0.035	0.186	4638	Tags = 58%, List = 19%, Signal = 71%
HALLMARK_G2M_CHECKPOINT	165	−0.58	−1.6	<0.001	0.041	0.071	1936	Tags = 27%, List = 8%, Signal = 29%
HALLMARK_MYC_TARGETS_V1	167	−0.58	−1.58	0.001	0.027	0.091	1773	Tags = 26%, List = 7%, Signal = 28%

### 3.6 Protein–protein interaction network analysis

**FIGURE 7:**
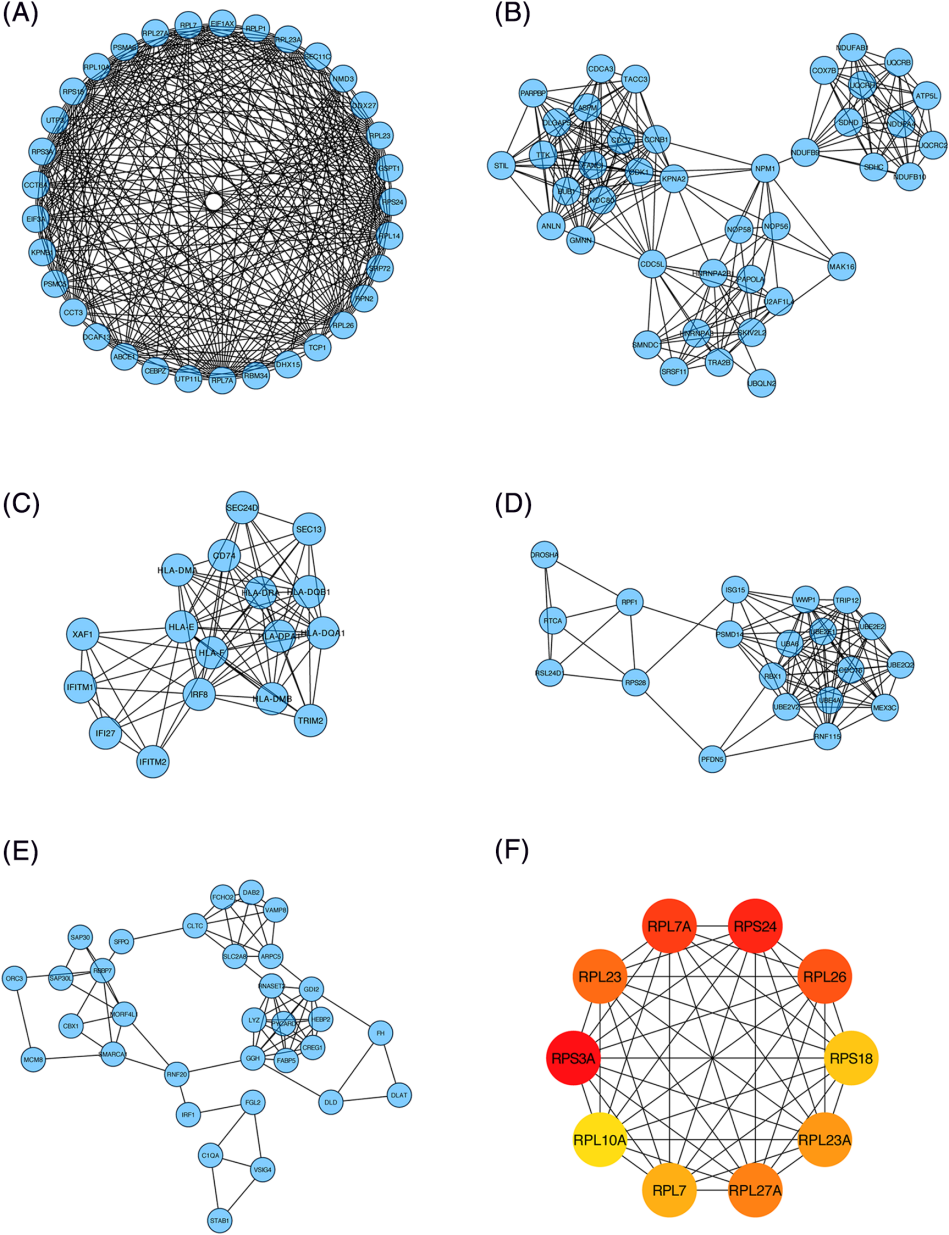
Visualization of protein–protein‐interaction networks of significant differential expressed genes (DEGs) based on the expression of PSMD14. (A–E) Five subnetworks identified by the MCODE algorism. (F) Ten hub genes of significant DEGs identified by PPI networks

To further understand the potential relationships between the DEGs associated to PSMD14 expression level, we performed PPI analysis (Figure 7A–E). The PPI network revealed that these DEGs were involved in five different subnetworks. Ten hub genes were identified by using CytoHubba. Interestingly, these genes were all ribosomal genes: RPL7A, RPS24, RPL26, RPS18, RPL23A, RPL27A, RPL7, RPL10A, RPS3A, and RPL23. This was consistent with the GO enrichment analysis (Figure 5). The PPI network within the 10 hub genes further proved the interaction between these proteins.

## 4 DISCUSSION

Survival rate of cancer patients has increased significantly in the recent decades benefitting from the advance in cancer research. However, despite the advance of osteosarcoma treatment in the recent decades, the survival rate has not been increased much.^4^ Discovering the mechanisms of malignancy, metastasis, prognosis markers, and therapeutic targets is one of the ways lead us to better treatment of osteosarcoma and potentially increase survival rate. In this study, we utilized the gene expression datasets to identify potential prognosis marker(s), and/or therapeutic targets. We utilized existing datasets to identify the DEGs between osteosarcoma and normal tissue or cells counterpart. Among the top DEGs, PSMD14 was the most significant one. By further analyzing the data, we found that high expression of PSMD14 was significantly correlated to higher osteosarcoma grade and metastasis (Figure 3). This data indicated that PSMD14 could potentially serve as a prognosis marker.

Ubiquitination and deubiquitination are balanced in the cells in order to maintain the proper levels of proteins controlled by ubiquitin‐dependent proteolysis. Deubiquitination of substrates is a way of recuing proteins from irreversible degradation, since ubiquitination is a modification for protein degradation. In eukaryotic proteasomes, several deubiquitinases were identified, such as PSMD14 (also named Poh1/Rpn11),^37^, ^38^ Usp14/Ubp6,^39^, ^40^ and Uch37.^41^ PSMD14 is a subunit of the 19S regulatory particle with the capacity of removing ubiquitin from proteasome substrates.^37^, ^38^, ^42^ The PSMD14 subunit is a metalloprotease that specifically cleaves “Lys‐63”‐linked polyubiquitin chains within the complex.^43^ PSMD14 is directly or indirectly involved in many cell processes because of its functional association with proteasome. Recently, PSMD14 has been shown to play role in hepatocellular carcinoma,^44^ breast cancer,^45^ and prostate cancer.^46^ Therefore, it would worthwhile to further investigate whether PSMD14 plays any roles in osteosarcoma cell processes, and whether PSMD14 can serve as an osteosarcoma therapeutic target.

CD8+ T lymphocytes are cytotoxic T lymphocytes. Infiltration of CD8+ T lymphocytes usually are associated with improved clinical outcomes in many different types of cancers.^47^ Interestingly, T lymphocytes are the one of major immune cells infiltrated in osteosarcoma tissues,^48^, ^49^ and high CD8+ T lymphocytes/FOXP3 cells ratio predicted better prognosis.^48^ However, by identifying from the gene expression, it seemed that CD8+ T lymphocytes were relatively abundant in the PSMD14 high expression osteosarcoma tumors (Figure 4). The status of the CD8+ T lymphocytes within these tumors will be very interesting to be further investigated. For instance, the possibility that PSMD14 high expression tumor cells could induce CD8+ T lymphocytes senescence or exhaustion could be an explanations for the positive correlation between CD8+ T lymphocytes, PSMD14 high expression and worse prognosis in osteosarcoma. In fact, the low PSMD14 expression osteosarcoma group, which had better prognosis, showed relatively enriched immunresponse as shown in Figure 6A–D, and showed higher apoptosis (Figure 6F). However, the DEGs in the higher PSMD14 expression group indicated more enrichment of cell proliferation genes, such as G2M checkpoints and Myc targets (Figure 6O,P). Whether high expression of PSMD14 osteosarcoma cells suppress immune cells activation and the possible cellular and molecular mechanisms warrant further investigation, which will potentially lead to better understanding of the mechanisms of osteosarcoma progression and/or metastasis or even more possible osteosarcoma treatments.

Taken together, in this study we reported that PSMD14 was significantly up‐regulated in osteosarcoma compare to normal tissue in all datasets we analyzed. The high expression level of PSMD14 was found to be correlated with metastasis and worse prognosis in osteosarcoma. Therefore, it is possible that PSMD14 can serve as a prognosis marker and a therapeutic target. To our knowledge, this is the first report demonstrating that PSMD14 can be potentially used as a prognosis marker for osteosarcoma. However, due to the limitation of bioinformatics analysis, the protein level of PSMD14 in osteosarcoma is not well studied. Our next goal is to confirm the protein level expression of PSMD14 in human osteosarcoma biopsies, and to further prove the possibility of PSMD14 as a prognosis marker and/or therapeutic target.

## CONFLICT OF INTEREST

The authors declare no potential conflict of interest.

## AUTHOR CONTRIBUTIONS

Both authors had full access to the data in the study and take responsibility for the integrity of the data and the accuracy of the data analysis. Conceptualization, Y.G. and Z.W.; Methodology, Y.G. and Z.W; Investigation, Y.G. and Z.W; Formal Analysis, Y.G. and Z.W; Resources, Y.G.; Writing ‐ Original Draft, Y.G. and Z.W; Writing ‐ Review & Editing, Y.G. and Z.W.; Visualization, Y.G. and Z.W; Supervision, Y.G.; Funding Acquisition, Y.G.

## ETHICAL STATEMENT

There was no human subject involved in this study.

## Data Availability

All datasets downloaded are listed in the Material and Methods section. All data and methods required to confirm the conclusions of this work are within the article, figures, and tables.
